# INTERGENERATIONAL HEALING: GENERATIVITY AND EMOTIONAL WELL-BEING AMONG ALASKA NATIVE ELDERS

**DOI:** 10.1093/geroni/igae098.1126

**Published:** 2024-12-31

**Authors:** Jordan Lewis, Steffi Kim, Lena Thompson

**Affiliations:** University of Alaska Fairbanks, College of Indigenous Studies, Fairbanks, Alaska, United States; University of Alaska, Anchorage, Alaska, United States; University of Minnesota Duluth, Duluth, Minnesota, United States

## Abstract

The concept of generativity, defined as teaching and guiding the next generation, is an important stage of human development. Little research has been conducted with Alaska Natives on their understanding of generativity and its role and impact on their emotional health. This presentation is part of a parent study that consists of 162 qualitative interviews with AN Elders over the course of 16 years and were thematically analyzed to explore generativity and emotional health in successful aging. Across the lifespan, Alaska Native Elders engage in healthy and unhealthy behaviors which may or may not contribute to a healthy identity, or their ability to age successfully. This presentation will discuss four cultural factors, or indigenous cultural generative acts, used by Alaska Native Elders to improve their emotional wellbeing and fill the role of an Elder in their family and community. Throughout this study, Elders described ways that they “found balance” in their lives, including through family influence, being recognized as respected Elders in their communities, community and cultural engagement, and spirituality or living their Native ways of life. Elders felt that it was their responsibility and role to share their wisdom, knowledge, and experiences to help younger generations avoid some of the challenges they experienced and live healthy lives. This presentation will discuss how cultural factors can be used to guide development of culture-specific mental health treatment approaches, and more broadly, prevention strategies for all ages. Intergenerational healing can result in improved mental health for Indigenous Peoples of all ages.

